# Collagen cross-linkage with riboflavin by Dr. Vinay Agarwal

**DOI:** 10.4103/0301-4738.60068

**Published:** 2010

**Authors:** Quresh B Maskati

**Affiliations:** Consultant, Maskati Eye Clinic, Mumbai - 400 004, India

Dear Editor,

I read with interest the article by Agrawal in the March issue of your journal.[1] It suffers from the following inconsistencies, in my opinion.

1. In the abstract it is mentioned that 68 eyes of 41 patients were included in this study. However, in the results section of the main text, it is mentioned that only 37 eyes of 25 patients were included. Perhaps the author meant that 37 eyes were analyzed, but 68 eyes were ‘included’. However, what is the purpose of inclusion if no analysis was done?
2. In the abstract it is also mentioned “All eyes completed was 12 months of follow-up and 37 eyes had one year follow-up” This sentence is grammatically incorrect and does not convey any meaning.
3. In the main text of the ‘Material and methods’ section, the topical anesthetic used is incorrectly spelt. The correct generic name is Proparacaine. Why was the manufacturing company name not mentioned? This is inconsistent with the rest of the article, where drugs made by multinationals were mentioned with the manufacturers' names in parenthesis (immediate next para).
4. In the main text of the ‘Materials and methods’ section, why are generic names mentioned for antibiotics and steroids and the trade name mentioned for an ocular lubricant?
